# Using inferior epigastric vascular anatomical landmarks for anterior inguinal hernia repair

**DOI:** 10.1186/s12893-019-0627-0

**Published:** 2019-10-29

**Authors:** Weiming Li, Yijun Li, Lili Ding, Xiongzhi Chen, Qingwen Xu, Shumin Li, Pengyuan Xu, Dali Sun, Yanbo Sun

**Affiliations:** Department of Gastrointestinal Surgery, Second Affiliated Hospital of Kunming Medical University, 374 Dianmian Road, Kunming, 650101 Yunnan Province China

**Keywords:** Inguinal hernia, Hernia repair, Vascular anatomical landmark

## Abstract

**Background:**

Inferior epigastric vascular anatomical landmarks for anterior inguinal hernia repair is an alternative surgical procedure. We present our experience and outcome of the way.

**Methods:**

We performed a retrospective analysis of 230 patients who received anterior tension-free hernia repair between May 2016 to May 2017. Among these cases, 120 were performed using the traditional transinguinal preperitoneal (TTIPP) technique while 100 were performed using the vascular anatomic landmark transinguinal preperitoneal (VALTIPP) technique. Between these two groups, we compared the operation time, length of hospital stay, complication rates, and the visual analog scale (VAS) for pain at 2 days, 3 months, and 6 months after surgery.

**Results:**

Surgery was well-tolerated in both groups with no significant hemorrhage or complications. The operation times for the VALTIPP and TTIPP groups were 42.52 ± 9.15 and 53.84 ± 10.64 min (*P* < 0.05), respectively. Ten patients in the VALTIPP group and 17 patients in the TTIPP group reported sensations of foreign bodies (*P* < 0.05). The VAS pain score in VALTIPP patients at 2 days (4.0 ± 0.5), 3 months (1.0 ± 0.3), and 6 months (0.9 ± 0.3) were significantly lower when compared with those of TTIPP patients (5.3 ± 0.9 at 2 days, 1.8 ± 0.4 at 3 months, and 1.1 ± 0.1 at 6 months, *p* < 0.05). No statistically significant differences were found in age, gender, BMI, hernia type and location, follow-up period, incidence of post-operative seromas, recurrence rate, or length of hospital stay.

**Conclusion:**

Anterior inguinal hernia repair using inferior epigastric vascular anatomical landmarks may lead to reduced operation times, reduced sensations of foreign bodies, and reduced post-operative pain. This technique is simple, practical, and effective in the management of inguinal hernias.

Inguinal hernias are common pathologies with high incidence. Tension-free inguinal hernia repair is currently the primary treatment for approximately 90–95% of abdominal hernias [1]. With the continuous innovation of surgical methods, endoscopic techniques are the preferred method in treating inguinal hernias [2–5]. However, no single surgical technique is suitable for all patients. In elderly patients with multiple comorbidities, open repair under local anesthesia may be a suitable surgical option [6]. With open surgery, tension-free hernia repair through the preperitoneal space approach is an ideal procedure. However, entering the preperitoneal space has been a challenge, and the neck-shoulder technique has been used in recent years. However, multiple technical challenges have yet to be resolved. For example, entry into the preperitoneal space may limit the use of the neck-shoulder technique if scarring at the neck of the hernia sac is encountered. Additionally, there is a risk of peritoneal rupture. At times, the preperitoneal exposure is inadequate, resulting in an inability to flatten the surgical mesh, resulting in nerve compression and pain.

In recent years, we have used the inferior epigastric vessel as anatomical landmarks in guiding efficient and accurate access into the preperitoneal space without being affected by scarring at the neck of the hernia sac. We were able to obtain complete coverage of the myopectineal orifice with a flattened surgical mesh without the need for a reinforcing mesh on the posterior wall of the inguinal canal, reducing the likelihood of nerve irritation and associated pain. This technique is currently used for tension-free inguinal hernia repair. In this retrospective analysis, we report patients who underwent preperitoneal access for inguinal hernia repair. We compared the operation times, length of hospital stays, complication rates, incidence of pain, and recurrence rates for the inferior epigastric vascular anatomical landmark technique versus the traditional neck-shoulder technique. The goal of this study is to explore the feasibility and advantages of using inferior epigastric vascular anatomic landmarks in anterior tension-free inguinal hernia repair.

## Materials and methods

### Patient selection

Our retrospective analysis includes 230 patients who received anterior tension-free inguinal hernia repair between May 2016 and May 2017. All the patients were at the Second Affiliated Hospital of Kunming Medical University. This study was approved by the Ethics Committee of the Second Affiliated Hospital of Kunming Medical University. Pain was measured by VAS (range: 0–10), a 10 cm line was drawn and marked equidistant 1–10, with 0 represents no pain and 10 representing the most severe pain [7]. The degree of pain was assessed at 2 days, 3 months and 6 months after surgery. Patients’ age, sex, body mass index (BMI), site of lesion, hernia type, operation time and postoperative hospital stay are shown in Table 1. All statistical analyses were performed with SPSS 17.0 software (SPSS,Chicago,IL,USA), *P* < 0.05 was considered significant.

**Table 1:** Patient demographic characteristics

Characteristics	TTIPP group	VALTIPP group	*P*-value
Cases (n)	120	110	0.69
Hernias (n)	126	118	0.58
Age, years	69.5 ± 5.3	69.8 ± 6.1	0.325
Men/women (n)	112/8	105/5	0.612
BMI (kg/m^2^)	23.5 ± 2.3	24.1 ± 2.5	0.156
Site of lesion (n)			
Unilateral/bilateral	114/6	102/8	0.531
Right/left	70/44	63/39	0.645
Type of hernia (n)			
Direct	78	73	0.368
Indirect	35	31	0.412
Femoral	3	4	0.172
Combined	10	10	0.238
Mean operation time, mean ± SD (min)	53.84 ± 10.64	42.52 ± 9.15	0.028
Postoperative hospital stay, (days)	2 ± 0.52	2 ± 0.37	0.165

*BMI* body mass index, *TTIPP group* Traditional transinguinal preperitoneal, *VALTIPP group* Vascular anatomical landmark transinguinal preperitoneal group

### Surgical technique

#### Preoperative preparation

Prior to surgery, patients fasted from solid food for 4 h and from liquids for 2 h, received skin preparation, and had their intended operative site marked by the surgeon. The elderly and patients with diabetes received pre-operative antibiotic prophylaxis. Surgery was performed by the same senior professional doctor in both groups.

#### Mesh

For patients in both groups, Easyprosthes Repair Patch (China) was used. The underlayer of this patch was circular with a diameter of 10 cm.

#### Surgical technique

**Fig. 1:**
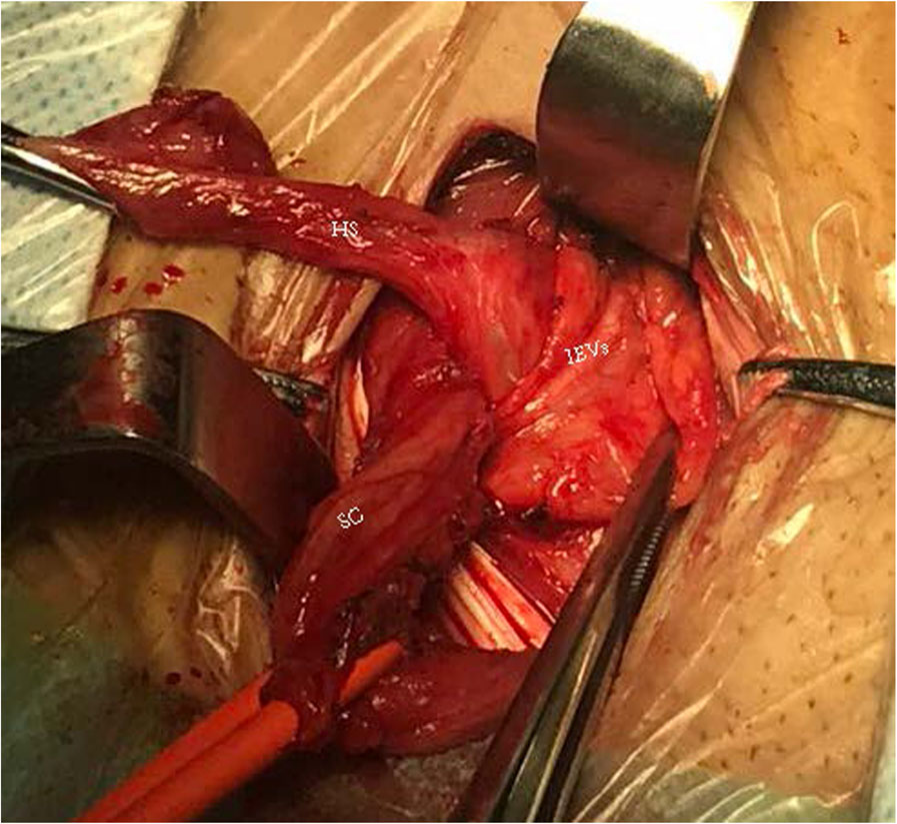
The inferior epigastric vessels (IEVs) as an anatomical landmark for entering the preperitoneal space. SC: spermatic cord, HS: hernial sac

**Fig. 2:**
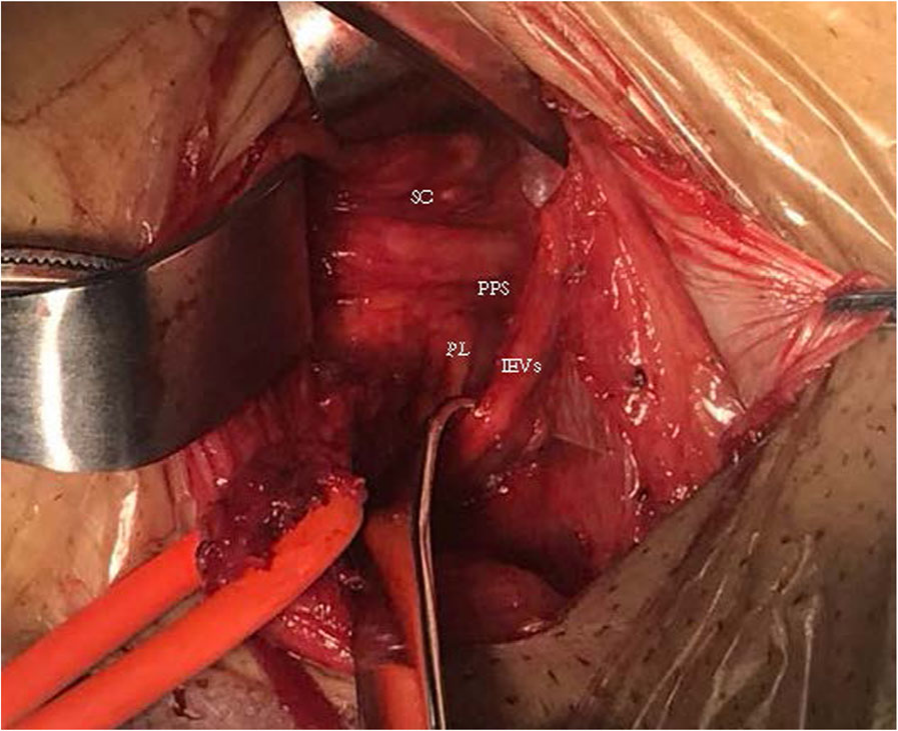
Abdominal wall and preperitoneal space. SC: spermatic cord; PL: public ligament; PPS: preperitoneal space

**Fig. 3:**
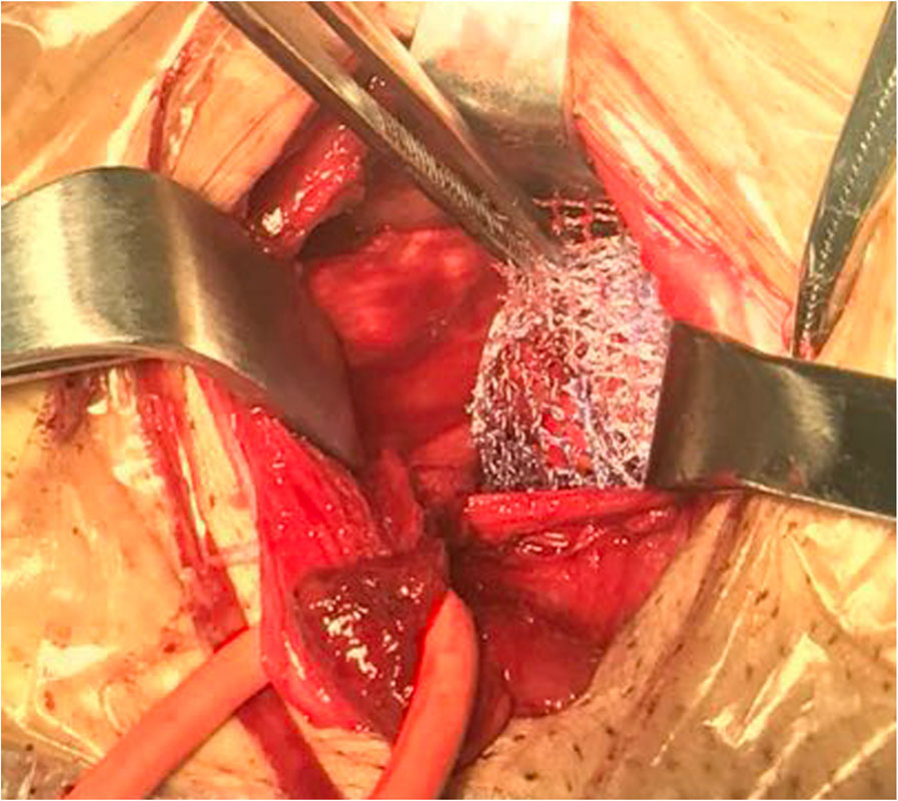
Flat mesh placement into the preperitoneal space

For the vascular anatomical landmark group, Local anesthesia was used in all operations,0.5% lidocaine 20 ml was administered as local anesthetic to the inguinal region [8]. An oblique incision was made by incising through the skin, subcutaneous tissue, and the aponeurosis of the external oblique muscle. Care was taken to protect the iliohypogastric nerve and ilioinguinal nerve. The spermatic root was then freed and retracted. The anterior and posterior layers of the transversalis fascia were then opened along the inferior epigastric vessel inside the inner ring. Entering the preperitoneal space, the hernia sac was located anterior to the spermatic cord. Straight or small sacs were retracted. Large hernia sacs were opened with the proximal end suture closed and distal end left open. In the preperitoneal space, the index finger of the surgeon was usually used for blunt separation of tissue. The Retzius and Bogros gap were usually separated with a wet gauze. Following best practices, the separation reached the pubic symphysis medially and the psoas muscle laterally, at least 3 cm superior to the joint muscle, 2 cm infero-medial to the inguinal ligament, and 6 cm infero-lateral to the spermatic cord. This ensured that the mesh could be flattened and carefully placed. The mesh was completely flattened manually and placed into the preperitoneal space. Posterior to the mesh is the hernia sac, while superior is the abdominal spermatic cord. Following best practices, the mesh completely covered the pubic muscular aperture, the inferior aspect of the pubic symphysis, superior to the inner ring by 3 cm, medial to the rectus abdominis, and lateral to the inguinal ligament. The mesh was secured to the abdominal fascia opened through the inner ring using absorbable sutures, preventing movement of the mesh. No mesh was placed in the posterior wall of the inguinal canal. The abdominal wall was subsequently closed (Figs.1, 2, and 3).

For the TTIPP group, Local anesthesia was used in all operations, the spermatic cord was first freed. The cremaster muscle and spermatic fascia were then longitudinally dissected along the spermatic cord. The hernia sac was then separated and retracted. Straight or small hernia sacs were directly returned to the peritoneal cavity. Large hernia sacs were opened and repaired proximally, with the distal segment left open. The peritoneal fascia was then opened at the neck-and-shoulder region of the hernia sac, revealing extraperitoneal fat and entering the preperitoneal space. Using a gauze wrapped around the surgeon’s finger, the preperitoneal space was bluntly dissected. The mesh was then placed and the inner ring was reconstructed. A flat mesh was secured to the posterior wall of the inguinal canal behind the spermatic cord. The abdominal wall was then sutured closed.

## Results

From May 2016 to May 2017, 230 patients received open repair for inguinal hernias. Between the two groups, no differences were found in sex, age, BMI, length of hospital stay, hernia type, and hernia location. Reduced operation times were observed for the VALTIPP group (42.52 ± 9.15 min) when compared to the TTIPP group (53.84 ± 10.64 min, *P* < 0.05) (Table 1).

The mean follow-up duration was 12.8 months for the VALTIPP group and 12.5 months for the TTIPP group, with no significant difference. No patients experienced recurrence or wound infection in either group. In the VALTIPP group, six patients were diagnosed with scrotal seroma by post-operative day 7, with complete re-absorption by 1 month in five patients. Fine-needle aspiration was performed in the remaining patient with complete resolution at 3 months follow-up. In the TTIPP group, 8 patients had scrotal seroma by post-operative day 7 with complete resolution in 6 patients by 1 month. Fine needle aspiration performed in 2 patients resulted in complete resolution by 3 months. Sensations of foreign bodies were experienced by 10 patients (9%) in the VALTIPP group and 17 patients (14.2%) in the TTIPP group (*P* = 0.038). Follow up time and complications are shown in Table 2.

**Table 2:** Comparison of complications between the two groups

	TTIPP group (*n* = 120) (%)	VALTIPP group (*n* = 110) (%)	*P*-value
Follow-up (months) mean ± SD	12.5 ± 2.4	12.8 ± 2.9	0.72
Recurrence (n)	0	0	NS
Seroma			0.170
7 days	8 (6.7%)	6 (5.5%)	0.72
1 month	2 (1.7%)	1 (0.9%)	NS
3 months	0	0	NS
Wound infection (n)	0	0	NS
Foreign body sensation (n)	17 (14.2%)	10 (9%)	0.038

*SD* standard deviation, *NS* not significant, *TTIPP Group* traditional transinguinal preperitoneal, *VALTIPP Group* vascular anatomical landmark transinguinal preperitoneal group

All patients underwent postoperative pain assessment using VAS. At 2, 3, and 6 months after surgery, patients in the VALTIPP group had VAS pain scores of 4.0 ± 0.5, 1.0 ± 0.3, and 0.9 ± 0.3, respectively, which were significantly lower than those of the TTIPP group (5.3 ± 0.9 at 2 months, 1.8 ± 0.4 at 3 months, and 1.1 ± 0.1 at 6 months, *P* < 0.05) (Table 3).

**Table 3:** Comparison of post-operative pain scores between the two groups

Time	Mean ± SD (range)		*P*
	TTIPP group (*n* = 120)	VALTIPP group (*n* = 110)	
2 days	5.3 ± 0.9	4.0 ± 0.5	0.03
3 months	1.8 ± 0.4	1.0 ± 0.3	0.02
6 months	1.1 ± 0.1	0.9 ± 0.2	0.01

*SD* standard deviation, *TTIPP group* traditional transinguinal preperitoneal, *VALTIPP group* vascular anatomical landmark transinguinal preperitoneal group

## Discussion

Since the introduction of tension-free hernia repair in the 1980s, traditional hernia repair under tension has been completely replaced due to rapid postoperative recovery, low recurrence, and low complication rates [9]. In open surgery, tension-free exposure of the preperitoneal space is ideal. To gain access to the preperitoneal space, the neck-shoulder technique may be used, albeit with challenges.

Recent studies in abdominal anatomy suggest the transversalis fascia is composed of two layers; separating these two layers is difficult and may lead to excessive bleeding. Prior to opening the peritoneum, the preperitoneal space of the inguinal hernia repair should be located between the peritoneum and the deep layer of the transversalis fascia, emphasizing the importance of surgical anatomy. We used the inferior epigastric vessel as our landmark. Anterior to this vessel is the superficial layer of the peritoneal fascia, and deep to this vessel is the deep layer of the transversalis fascia. During surgery, the deep and superficial layers of the peritoneal fascia were opened along the inferior epigastric vessel, effectively accessing the preperitoneal space. This technique is not affected by scarring at the hernia neck or by variations in anatomy. Additionally, this creates ample surgical exposure with minimal nerve and vessel intrusion. If the surgical exposure is properly identified, damage to vessels or the spermatic cord will be dramatically reduced. This allows for uneventful implantation of the mesh with complete coverage of the myopectineal orifice. These principles are consistent with anatomic biomechanics. Lastly, a reinforcement mesh on the posterior wall of the inguinal canal is not required, thereby avoiding manipulation of the inguinal nerve.

The incidence of chronic pain after tension-free inguinal hernia repair is approximately 10–30% [10–18], a major cause for concern. Based on our retrospective analysis, the VAS scores of the VALTIPP patients were significantly lower than those of the TTIPP patients at 2 days, 3 months, and 6 months after surgery. This suggests the identification of the preperitoneal space using the inferior epigastric vessel as landmarks can significantly reduce the incidence of post-operative pain after inguinal hernia repair, possibly due to reduced contact between the mesh and nerves. The incidence of sensation of a foreign body was significantly lower in the VALTIPP patients than in the TTIPP patients, as there was no reinforcement mesh placed in the posterior wall of the inguinal canal, thereby reducing nerve irritation and scar formation. Even though the VALTIPP patients were not implanted with a reinforcing mesh on the posterior wall of the inguinal canal, they were not found to have a higher incidence of recurrence at follow-up, The follow-up time is limited and the clinical effect needs further observation. Our usage of vascular anatomical landmarks allowed for the effortless identification of both layers of the transversalis fascia, allowing quick and accurate entry into the space between the peritoneum and the transversalis fascia. This reduces the difficulty in locating the preperitoneal space and lowers the risk of peritoneal rupture. No reinforcing mesh was placed on the posterior wall of inguinal canal. These two reasons led to shorter operation time, as demonstrated in the VALTIPP patients in this study. Scrotal seroma is also a common post-operative finding, occurring in 7 patients throughout the study (5–7%), with no significant difference between the two groups. In most cases, the seroma resorbed spontaneously, but fine-needle aspiration may be attempted if unresolved at 1 month after surgery.

## Conclusions

For these reasons, using inferior epigastric vascular anatomical landmarks, as opposed to traditional surgery, for anterior inguinal hernia repair, may significantly reduce pain and sensations of foreign bodies. Additionally, operation time is shortened with good safety and efficacy profiles, and can be performed under local anesthesia.

## Data Availability

The datasets used and/or analysed during the current study are available from the corresponding author on reasonable request.

## Abbreviations

BMI: Body mass index
CT: Computerized tomographic scanning
HS: Hernial sac
IEVs: Inferior epigastric vessels
PL: Public ligament
PPS: Preperitoneal space
SC: Spermatic cord
TTIPP: Traditional transinguinal preperitoneal
VALTIPP: Vascular anatomic landmark transinguinal preperitoneal
VAS: Visual analog scale
